# What Have Slow Progressors Taught Us About T1D—Mind the Gap!

**DOI:** 10.1007/s11892-019-1219-1

**Published:** 2019-09-10

**Authors:** Kathleen M. Gillespie, Anna E. Long

**Affiliations:** Diabetes and Metabolism, Bristol Medical School, University of Bristol, Level 2, Learning and Research, Southmead Hospital, Bristol, BS10 5NB UK

**Keywords:** Type 1 diabetes (T1D), Slow progression, Adult onset, Islet autoantibodies

## Abstract

**Purpose of Review:**

Progression rate from islet autoimmunity to clinical diabetes is unpredictable. In this review, we focus on an intriguing group of slow progressors who have high-risk islet autoantibody profiles but some remain diabetes free for decades.

**Recent Findings:**

Birth cohort studies show that islet autoimmunity presents early in life and approximately 70% of individuals with multiple islet autoantibodies develop clinical symptoms of diabetes within 10 years. Some “at risk” individuals however progress very slowly. Recent genetic studies confirm that approximately half of type 1 diabetes (T1D) is diagnosed in adulthood. This creates a conundrum; slow progressors cannot account for the number of cases diagnosed in the adult population.

**Summary:**

There is a large “gap” in our understanding of the pathogenesis of adult onset T1D and a need for longitudinal studies to determine whether there are “at risk” adults in the general population; some of whom are rapid and some slow adult progressors.

## Introduction

Type 1 diabetes (T1D) results from a breakdown in immune regulation that leads to expansion of autoreactive B cells as well as CD4+ and CD8+ T cells targeting the insulin-producing beta cells of the islets of Langerhans [1]. The humoral response results in circulating autoantibodies to islet antigens including insulin (IAA) [2], glutamic acid decarboxylase (GADA) [3], islet antigen-2 (IA-2A) [4], zinc transporter 8 (ZnT8A) [5] and more recently tetraspanin 7 (Tspan 7A) [6]. It has been known for some time that progression to clinical diabetes is not a linear process but proceeds at variable pace in different individuals [7]. von Herrath et al. [8] suggested a relapsing and remitting pattern caused by dynamic interactions between immune cells and beta cells. Prospective birth cohort studies show that autoantibodies can be detected in children at risk of T1D from 6 months of age with a peak in seroconversion between 2 and 3 years [9] and children who are multiple islet autoantibody positive early in life have a 70% risk of diabetes within 10 years and an 84% risk within 15 years [10]. Recent observations of enriched B cell infiltration in islets from young children diagnosed with diabetes under the age of 7 years [11] which was not observed in those developing the condition over the age of 13 years, with a heterogenous pattern in between, may suggest that the “later onset” pattern of insulitis would be detected in pancreas from slow progressors.

Our initial studies in the Bart’s Oxford (BOX) family study of T1D [12] suggested the presence of multiple islet autoantibody-positive individuals in whom progression to clinical diabetes was delayed. We therefore established the Slow or Non-progressive Autoimmunity to the Islets of Langerhans (SNAIL) cohort to understand better the natural history of autoimmunity in individuals we describe as “slow progressors” who do not develop clinical symptoms for more than a decade after detection of multiple islet autoantibody positivity [13••]. These individuals may be an example of a slow chronic autoimmunity, and we have hypothesised that this may be enabled through natural regulation of the autoimmune process in these individuals.

## SNAIL Participants

“At risk” individuals in SNAIL [13••] were derived from five international studies: BABYDIAB [14], the Diabetes Autoimmunity Study in the Young (DAISY) [15], All Babies in Southeast Sweden (ABIS) [16], the BOX Family Study [12] and the Pittsburgh Family Study [17]. The studies are united by their focus on the natural history of autoimmune diabetes. BABYDIAB, DAISY and ABIS included prospective follow-up of children from birth (blood samples were taken at 9 months or 1 year), while BOX and the Pittsburgh studies enrolled first degree relatives of individuals with diabetes throughout life.

To date, 132 participants have been identified as slow progressors who remained diabetes free for more than 10 years after multiple autoantibodies (mAabs) were first detected (Table 1). These slow progressors represent on average 30% of the autoantibody-positive individuals identified, but the frequency varies depending on when islet autoantibodies were first detected. Given the longitudinal nature of the studies in SNAIL participants continue to be followed (median 4 years, IQR 2–9 years). During follow-up, 42 of 132 slow progressors were diagnosed with T1D indicating that these individuals remain at high risk although 90 are diabetes free. It is important to note that in the birth cohorts, young children from BABYDIAB and DAISY with multiple autoantibodies were represented within the SNAIL population showing that slow progression is not an exclusive characteristic of age of first multiple islet autoantibody detection. Interestingly, ABIS has identified multiple antibody-positive individuals through antibody screening in the general population. In this study, however, only half of the high-risk children identified developed diabetes within 10 years of follow-up, suggesting that slow progressors could be more common in the general population than in those selected through genetic risk or family history. This may reflect reduced genetic risk and/or environmental exposures.

**Table 1:** Description of participants in the SNAIL study

	Overall	BABY DIAB	DAISY	ABIS	BOX	Pittsburgh
*n*	132	22	30	11	36	33
Age at first antibody test, median (IQR)	7 (1–18)	1	1 (1–2)	1	18 (13–38)	18 (11–33)^a^
Age at mAab+ sample, median (IQR)	10 (5–20)	5 (2–5)	7 (4–10)	5	18 (13–38)	18 (11–37)
Male, *n* (%)	69 (54)	16 (73)	17 (57)	7 (64)	15 (42)	17 (52)
Years of follow-up since mAab+ detection, median (IQR)	14 (12–19)	13 (11–14)	12 (11–13)	13 (13–14)	17 (14–24)	20 (13–26)
Diabetes free at follow-up, *n* (%)	90 (68)	12 (55)	23 (77)	10 (100)	22 (61)	23 (70)
Genetic data available	121	22	30	10	36	23

For birth cohorts BABYDIAB, DAISY and ABIS, the age at first antibody test represents the first early life sample available sample for analysis. For BOX and the Pittsburgh study, this represents the first available sample after recruitment and there is no information on the time of seroconversion for these individuals

*n* number, *IQR* interquartile range, *mAab+* multiple autoantibody positive

(Reproduced from: Long AE, et al. Diabetologia. 2018 61:1484–1490; 10.1007/s00125-018-4591-5; Creative Commons user license https://creativecommons.org/licenses/by/4.0/) [13]

^a^Earliest sample available

## Genetic Factors Affecting Rate of Progression

The effect of the HLA class II DRB1*04-DQB1*0302 (DR4-DQ8) and DRB1*03-DQB1*02 (DR3-DQ2) on increased risk of T1D is well established [18, 19], but given their role in antigen presentation, it has been suggested that these class II haplotypes are involved in the initiation of autoimmunity while HLA class I haplotypes drive subsequent beta cell destruction [20]. Independent genetic determinants of insulitis and diabetes have been identified in the NOD mouse [21], and it has been postulated in humans that HLA class I risk genes (for instance HLA A*24) define rate of progression [20] perhaps through effects on CD8+ T cells. In the DAISY study, however, the HLA class II DR3/4-DQB1*0302 genotype had a dramatic influence on both development of islet autoimmunity and progression to T1D and the *PTPN22(R620W) T* allele significantly influenced progression to persistent islet autoimmunity [22]. Analysis of progression in the T1D Prediction and Prevention study (DIPP) cohort suggested protective effects of the A*03 allele while the B*39 was associated with seroconversion from one to two islet autoantibodies [23]. In BABYDIAB, islet autoantibody-positive children with the rs2111485 GG genotype in the T1D-associated viral-response gene, interferon-induced helicase C domain-containing protein 1 (IFIH1), progressed more quickly to diabetes (31% within 5 years) compared with children carrying the GA or AA genotypes (11% within 5 years) [24]. This suggests interaction between genetic and environmental determinants of T1D. There is also a suggestion of direct effects of common genetic variants associated with T1D on immune cell function; for instance, the IL2/IL2-R signalling pathway confers decreased ability to respond to IL2 with a resultant relative reduction in suppressive Treg function [25]. Genetic variants in PTPN2 may contribute to this.

## Genetic Risk in SNAIL Participants: Slow Progressors Have Less HLA-Mediated Genetic Risk than Individuals Diagnosed in Childhood

HLA class II *DQB1* risk genotypes were available from 121 slow progressors in SNAIL in the format DQB1*0201/DQB1*0302 (DQ2/DQ8). The high-risk combination was decreased (28% vs 42%) while intermediate risk genotypes were more common (55% vs 49%) when compared with 348 children from BOX diagnosed under 5 years of age, who were designated rapid progressors (*p* = 0.011, Table 2). Nevertheless, the slow progressors are a relatively high-risk group as their genetic risk profile was similar to that observed in 1217 BOX participants diagnosed between 10 and 20 years of age (DQ2/DQ8, 26%). Only two of 121 (1.7%) slow progressors carried the protective DQB1*0602 allele, the same as the proportion found in rapid progressors (1.7%) [13]. HLA DRB1*04 subtypes are also an important consideration because haplotypes containing DRB1*0403 and *0407 are protective [19]. In the BOX slow progressor cohort, of 22 individuals positive for HLA DRB1*04, subtype data were available for 19; 13 were positive for *0401, 4 for *0404, 1 for *0405 and 1 for *0408, all susceptible subtypes.

**Table 2:** The frequency of high-risk HLA genotypes by age at onset in the BOX study compared with slow progressors in the SNAIL study

	Diagnosis age (years)	High risk (DQ2/DQ8)	Intermediate risk (either DQ2 or DQ8)	Low risk (not DQ2/not DQ8)
BOX probands	Under 5 (%)	42.2	49.1	8.6
	5–9 (%)	34.9	54.7	10.4
	10–14 (%)	30.5	55.4	14.1
	15–20 (%)	19.4	62.8	17.8
SPs	N/A	28.9	55.4	15.7

Increasingly, measurements of genetic risk have moved away from HLA to use of simplified composite genetic risk scores where HLA and non-HLA risk are combined quantitatively using T1D-associated SNPS. The genetic risk scores (GRS) are weighted by odds ratio and can be useful to help classify diabetes clinically [26, 27]. Specificity and sensitivity testing in the Environmental Determinants of Diabetes in the Young (TEDDY) study show that composite scores (especially those striving to account for the complexity of the HLA) improve genetic risk assessment [28]. As GRS are based on genome-wide association studies (GWAS) which were largely carried out on children from European populations or populations of European extraction, broader GWAS are required to inform risk in other populations [29].

Although most studies of the genetics of T1D have focused on childhood onset diabetes, recent studies in UK Biobank of all diagnoses of diabetes using a T1D GRS [30••] showed that T1D occurs at a consistent rate in each decade of life. Indeed, as the GRS are based on GWAS data on children diagnosed with diabetes under the age of 15 years when T1D susceptibility genes are enriched, it is likely that the frequency of adult onset T1D has been underestimated using this approach. Nevertheless, this study shows that half of autoimmune T1D is diagnosed over the age of 30 years. This begs the question “where do they come from?” or “when was islet autoimmunity triggered in these patients who are diagnosed as adults?”

## Mind the Gap!

**Fig. 1:**
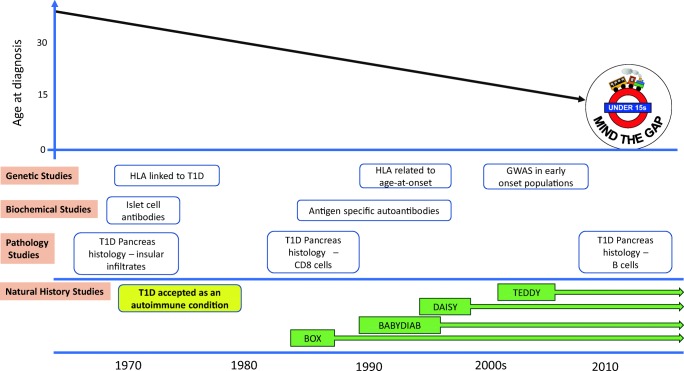
Timeline towards focused study of type 1 diabetes in childhood populations. In the 1970s, data from pancreatic histology, the description of islet antibodies and genetic association with HLA confirmed the autoimmune basis of the condition. The observation of increased HLA mediated susceptibility in those developing the condition young, and the need for defined phenotypes for successful GWAS has resulted in excellent characterisation of early-onset diabetes but relative lack of understanding of the pathogenesis of adult onset type 1 diabetes

As represented schematically in Fig. 1, there is a large gap in our understanding of the pathogenesis of autoimmune diabetes in adulthood as the majority of previous research has focused on childhood onset (under the age of 15 years). Moreover, most birth cohorts do not have extended follow-up into adulthood.

Birth cohorts show that 84% of children who are positive for multiple islet autoantibodies early in life develop diabetes within 15 years. Slow progressors as defined in SNAIL can therefore account for only a small proportion of adult onset autoimmune diabetes. This leads to some questions we do not yet know the answer to:

- Do some individuals in the general population seroconvert to islet autoantibody positivity in late adolescence/adulthood or do they trigger autoimmunity in early age but this is more regulated and therefore progress more slowly, if at all?
- If the rate of onset of diabetes is the same in adulthood and childhood, are approximately one in three hundred of the adult UK population “at risk”?
- Do they develop multiple islet autoantibodies?
- Are there adults who progress rapidly from islet autoantibody positivity to clinical onset?
- Is the pathogenesis driven by GAD autoimmunity as described in cases of latent autoimmune diabetes of adults (LADA)?

## Islet Autoantibody Characteristics in Slow Progressors

Studies of neonatal diabetes show that most cases of diabetes diagnosed before 6 months are unlikely to be autoimmune, but the majority of those diagnosed after the age of 6 months have the genetic characteristics of T1D [31]. Islet autoantibodies are detectable by 5 years of age in most future childhood T1D cases [32], in many by 2 years of age [14], and IAA (autoantibodies to insulin, often the first to appear) have been detected as early as 6 to 12 months of age [33]. Indeed, a small proportion of islet autoantibody-positive children in the TEDDY study first developed evidence of islet autoimmunity at the age of 3 months [34]. IAA appearing early in life tend to be high affinity of the IgG1 subclass. Rapid spreading of the immune response to other islet autoantigens occurs in early onset cases while those who progress to clinical symptoms later in life more frequently have low-affinity autoantibodies and atypical epitope reactivities [35]. A study of progression in the US-based DAISY cohort showed that age at onset of diabetes is closely correlated with the age of appearance of the first islet autoantibodies and the level of antibodies to insulin, but not glutamic acid decarboxylase (GAD) or islet antigen 2 (IA-2) [36]. A follow-up study of the 118 multiple islet autoantibody-positive individuals in DAISY showed that islet autoantibodies appear later and at lower levels in the 27 slow progressors compared with those who progressed [37]. In the Belgian Diabetes Registry, the 20-year progression rate of multiple islet autoantibody-positive siblings and offspring under 40 years of age was reported [38••]. Their data mirrored the combined BABYDIAB, DAISY and DIPP birth cohort data [10] with the majority of multiple islet autoantibody-positive individuals developing diabetes within 20 years. Risk was not assessed in parents, and this may contribute to the difference in number of slow progressors observed as SNAIL includes parents as well as siblings and offspring of individuals with diabetes. The ethnic composition of most studies is predominantly Caucasian apart from DAISY which includes four Hispanic slow progressors. In the TEDDY study, where 8503 children were followed to age 6 (and more recently 8 years), an early peak of IAA only in 43% of children who seroconverted within the first year of life declined over the following 5 years and 38% had GADA only, which increased until the second year of life and remained relatively constant over the follow-up period [39, 40]. This suggests heterogeneity in early islet autoimmunity, but ultimately, there is epitope spreading in most childhood cases before diagnosis.

## Could There also Be Heterogeneity in Adult Onset T1D? What Are the Islet Autoantibody Patterns in Slow Progressors?

All SNAIL participants had, by definition, multiple islet autoantibodies [13••], but the autoantibody patterns varied between cohorts. The first antibodies detected in about two thirds of children in BABYDIAB were IAA. Loss of IAA has been associated with delayed progression [41••]. Further development of the algorithm used in this study to analyse longitudinal data from the TEDDY cohort identified clusters with stratified risk profiles varying from 6 to 84% risk of progression within 5 years [42••]. In the SNAIL cohorts, IAA were more common in the first mAab positive samples from BABYDIAB and ABIS children. Unexpectedly despite most being tested first as adults, half of BOX and Pittsburgh family study participants were also IAA positive in their first sample. GADA are the first islet autoantibodies detected in about a third of children who develop diabetes, but are also prevalent in adult onset disease. GADA were common in all SNAIL cohorts, but overall were more frequent in older individuals. Antibodies that recognise IA-2 and ZnT8 are considered to develop later in the pathogenesis of T1D and are associated with progression. It is counterintuitive therefore that ZnT8A were the second most frequent antibody in slow progressors with no differences observed between autoantibodies to the ZnT8 R325W variants. In contrast, IA-2A were less common, and BABYDIAB participants had a particularly low prevalence of IA-2A (14%) in their first mAab sample. Indepth analysis of autoantibody epitopes and IgG subclasses in slow vs. rapid progressors is ongoing. During follow-up, however, 18 of 22 (82%) BABYDIAB SNAIL participants seroconverted to IA-2A positivity indicating that antigen spreading continued in these individuals despite slower progression.

## Latent Autoimmune Diabetes in Adults

The most common screening tool for LADA are GADA. An assay targeting terminally truncated (aa96-585) GAD [43] improved the clinical phenotyping of LADA and identified those with an increased need for insulin therapy [44]. What would a screen of the general adult population for IAA/truncated GADA/IA2-A and ZnT8A show? This will be an important focus for studies of adult onset T1D moving forward.

## Immune Cell Subsets

Upregulation of MHC class I on beta cells and insulitis dominated by CD8+ T cells are recognised as major determinants of beta cell destruction, and this process is variable both between and within pancreas samples [45–47]. Mechanistically, beta cell destruction can involve the release of cytolytic granules containing perforin and granzyme by CD8+ T cells or be mediated through Fas and Fas ligand-dependent interactions, while CD4+ T cells provide help. Measures of CD8+ T and CD4+ T function in at-risk individuals may therefore provide insights into slow progression. In addition, advances in the understanding of regulatory immune cell subsets have led to studies indicating that although regulatory T cells appear to be normal in number, individuals with diabetes have some functional defects in their regulatory T cells. These include a reduced capacity to respond to IL2 [25]. In addition, effector T cells in those who develop diabetes may be more resistant to regulation, as shown by a reduction in suppression of effector T cells by both naturally occurring T regulatory cells and in vitro-generated adaptive T regulatory cells [48] and diminished IL2 responsiveness in antigen-experienced CD4+ T cells [49]. A more recent report highlights the dynamic nature of immune cell subsets with distinct immune pheontypes arising at various stages before diagnosis in “at risk” individuals, and some of these are transient. For instance, changes in IL2 responsiveness precede or coincide with transiently altered B cell responses [50•]. This highlights the need for longitudinal follow-up of immune cell subsets in individuals with multiple islet autoantibodies, investigations which are currently ongoing in SNAIL.

## Conclusions

Apart from TrialNet and the Belgian Diabetes Registry, most longitudinal research studies in T1D have focused upon enrolling high-risk children at birth identified through family history of disease or genetic risk. This has led to limits in understanding the development of the disease in adults and the general population. Cross-sectional studies in adults have suggested that GADA are the most common islet autoantibody, but interpretation is limited by the different definitions of diabetes and durations of disease at sampling. In the UK, adult diabetes care is conducted by primary care physicians, and therefore, it is challenging to collect routine data from adults. Furthermore, the outdated islet cell autoantibody (ICA) assay is still often used routinely rather than antigen-specific assays for immunophenotyping/characterising individuals and this can make comparisons difficult. The Islet Autoantibody Standardization Programme (IASP) has identified high-quality assays including ELISAS, radioimmunoassays (RIA), luciferase immunoprecipitation system (LIPS) assays and agglutination PCR which are easier to perform than ICA assays and will provide improved characterisation of islet autoantibodies at diagnosis of T1D.

In addition, we have limited understanding of the factors that contribute to differences in incidence between countries and this may be exacerbated by the recruitment of individuals with similar high genetic risk for studies of diabetes. Cross-sectional screens of the general population in countries with differing incidence (Finland vs Russian Karelia [48] and the UK vs Lithuania [49]) suggested some geographical differences in islet autoantibody profiles in childhood. Follow-up of autoantibody-positive individuals in the general population from birth as well as extended follow-up of existing cohorts is warranted to establish answers to the questions set out in this review.

One unexpected outcome of our studies of slow progression to T1D is that it has highlighted the knowledge gaps in understanding the pathogenesis of the disease adult onset cases.

## Abbreviations

ABIS: All babies in Southeast Sweden
BOX: Bart’s Oxford family study
DAISY: The Diabetes Autoimmunity Study in the Young
GADA: Autoantibodies to glutamic acid decarboxylase
GRS: Genetic risk scores
GWAS: Genome-wide association studies
HLA: Human leucocyte antigen
IAA: Insulin autoantibodies
IA-2A: Autoantibodies to IA-2
LADA: Latent autoimmune diabetes in adults
IL2: Interleukin 2
mAabs: Multiple autoantibodies
ZnT8A: Autoantibodies to zinc transporter 8
